# Recognizing Subacute Combined Degeneration in Patients With Normal Vitamin B12 Levels

**DOI:** 10.7759/cureus.15429

**Published:** 2021-06-03

**Authors:** Brianna Burlock, Jason P Williams

**Affiliations:** 1 Neurology, Emory University School of Medicine, Atlanta, USA; 2 Internal Medicine, Emory University School of Medicine, Atlanta, USA

**Keywords:** serum vitamin b12, subacute combined degeneration, methylmalonic acid, pernicious-anemia, cobalamin deficiency

## Abstract

Vitamin B12 deficiency is commonly associated with dementia in patients over the age of 65 years; however, it can affect people of all ages. Recognizing the clinical sequelae of subacute combined degeneration is essential for the timely diagnosis and treatment of vitamin B12 deficiency. In this report, we describe a case of a young man presenting with several months of neuropathy, depression, and abdominal symptoms. His initial vitamin B12 levels were within normal limits, but an elevated methylmalonic acid level and subacute combined degeneration of his spine on MRI confirmed the diagnosis of vitamin B12 deficiency. The patient later tested positive for autoantibodies associated with pernicious anemia. His symptoms improved with intramuscular injections of cyanocobalamin. This case highlights the importance of recognizing vitamin B12 deficiency in patients of all age groups even in the setting of apparently "normal" B12 levels.

## Introduction

Studies on vitamin B12 deficiency predominantly focus on individuals older than 65 years of age and populations that are vulnerable to malnutrition [1,2]. In older populations, vitamin B12 deficiency can present as dementia and is commonly screened for during a dementia workup. A vitamin B12 level screening is not routinely ordered in young patients with normal dietary habits. The prevalence of vitamin B12 deficiency is about 20% in industrialized countries [1]. In the Framingham study, researchers discovered that patients over the age of 65 had a 12% prevalence of vitamin B12 deficiency [3]. The researchers further found that elderly people with apparently "normal" vitamin B12 levels are often metabolically deficient in vitamin B12 [3].

Pernicious anemia is the most common etiology of vitamin B12 deficiency worldwide, causing 20-50% of the cases [4]. The overall prevalence is 0.1% in the general population, and even higher in those over 60 years of age (1.9%) [4]. Although this prevalence rate may not seem to indicate a heavy disease burden, many previous studies have postulated that the epidemiology reports are often not accurate due to multiple reasons including variance in vitamin B12 cut-off thresholds and underdiagnosis due to the condition's presentation in advanced stages [2,5]. The variable and subtle clinical manifestations of pernicious anemia make it difficult for clinicians to recognize the condition. However, the advanced form of the disease can have a profound impact on patients' quality of life and can lead to devastating long-term effects if left untreated. Prompt recognition and early intervention are critical to avoid permanent damage associated with pernicious anemia.

## Case presentation

A 33-year-old male with a past medical history of depression and gastroesophageal reflux disease presented to the emergency department with complaints of worsening weakness and falls for the past 11 months. He had initially noted numbness in his fingers about a year prior to the presentation, which had resolved spontaneously after one month. A few months later, the patient had developed bilateral foot numbness followed by progressively bilateral leg weakness and gait instability. This had significantly affected the patient's mobility leading to the resignation from his job, and he had become wheelchair-bound. These symptoms were associated with a decreased appetite, which had resulted in unintentional weight loss. He reported consuming meat and vegetables regularly. Additional review of systems revealed urinary incontinence, impotence, and fatigue. The patient denied abdominal pain, trauma, recent travel, sick contacts, toxin exposures, nausea, vomiting, diarrhea, fever, chills, headache, loss of consciousness, or vision and hearing changes. He lived with his wife who had been preparing his meals and helping him move around the house. He denied tobacco, alcohol, or recreational drug use.

On examination, the patient was alert and oriented to person, place, time, and situation. His recall memory was intact. Cranial nerves 2-12 were also intact. Strength in the deltoids and biceps were 5/5 bilaterally. Strength in the wrist extensors and flexors were 4/5, and finger abduction and adduction were 4/5 on the left and 4/5 on the right. Strength in the hip flexors was 3/5, knee extensors 4/5, foot dorsiflexion 4/5, and foot plantarflexion 4/5. Sensation examination showed loss of vibration and proprioception bilaterally up to the knee. There was also loss of sensation to pinprick and light touch to mid-thighs and decreased sensation in bilateral fingertips. His reflexes were decreased to 1+ at the patella, 0 at the ankles, and Babinski demonstrated upgoing toes. His coordination was intact in the upper extremities, but this could not be assessed in the lower extremities due to weakness. The patient’s skin was flaky and dry in all extremities.

**Figure 1:**
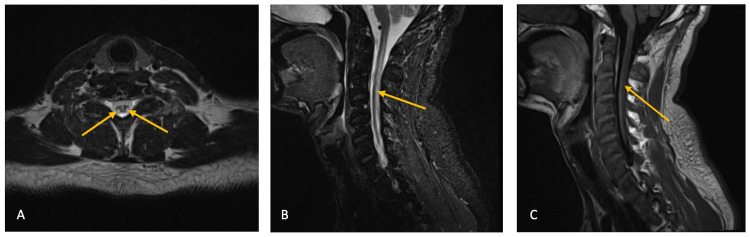
MRI of the cervical spine T2 sagittal (A) and T2 axial (B) MRI of the spine demonstrating symmetric bilateral high signal within the dorsal columns that is commonly described as the inverted "V" sign (arrows). T1 post-contrast image (C) confirmed that these were non-enhancing lesions (arrow) MRI: magnetic resonance imaging

The patient was admitted to the hospital for further workup. His laboratory panel was significant for macrocytic anemia with a hemoglobin of 11.2 g/dL (reference range: 13.7-17.5 g/dL) and a mean corpuscular volume (MCV) of 107.8 fL (reference range: 80-100 fL). Vitamin B12 level was normal at 229 pg/mL (reference range: 180-914 pg/mL) as was his folate levels at >23 ng/mL (reference range: >5.9 ng/mL). Given the macrocytic anemia and neurologic findings, homocysteine and methylmalonic acid levels were obtained, and both were found to be elevated at 190.1 umol/L (reference range: ≤11.4 umol/L) and 53,100 nmol/L (reference range: 87-318 nmol/L), respectively. MRI of the spine showed non-enhancing, non-expansile, increased T2 signal along the dorsal lateral margins of the cervicothoracic cord and dorsal columns of the cervical cord (Figure 1A-1C).

While awaiting these results, the patient was empirically started on 1,000 mcg of intramuscular cyanocobalamin daily, and his omeprazole was stopped. After seven days of daily injections, the frequency of intramuscular B12 supplementation was cut down to weekly for one month, and then to monthly. He was followed up in the clinic by neurology, gastroenterology, physical therapy, and his primary care provider. After he left the hospital, his serum antibody results were obtained, which confirmed the presence of intrinsic factor and parietal cell antibodies. Additionally, his outpatient gastroenterologist detected an elevated gastrin level of 603 pg/mL (reference range: <100 pg/mL), further supporting the diagnosis of autoimmune gastritis. After four months of treatment, the patient is now able to walk without an assistive device but still suffers from some mild weakness and sensory deficits. His homocysteine, methylmalonic acid, and vitamin B12 levels have all normalized.

## Discussion

Vitamin B12 measurement

Our patient was found to have "normal" vitamin B12 levels as per his laboratory results despite his experiencing severe neurologic consequences of B12 deficiency. The “normal” vitamin B12 assays could be attributed to the prevalence of high rates of false-negative results. Up to 50% of patients with clinical signs of vitamin B12 deficiency will have a "normal" value of over 200 pg/mL (Table 1) [6]. If you increase the threshold of "normal" to 350 pg/ml, the sensitivity improves but the specificity dramatically falls to 25% (Table 1). The patient’s macrocytic anemia and neurologic findings prompted testing for methylmalonic acid and homocysteine, which have much higher sensitivity for vitamin B12 deficiency [6].

**Table 1:** Laboratory testing for vitamin B12 deficiency MMA: methylmalonic acid

Tests for vitamin B12 deficiency	Sensitivity	Specificity
Serum vitamin B12 (<200 pg/mL)	50%	80%
Serum vitamin B12 (with the threshold of normal increased to <350 pg/mL)	90%	25%
Serum MMA (>400 nmol/L)	98%	Variable/unknown
Serum or plasma homocysteine (>21 µmol/)	96%	Unknown

Subacute combined degeneration

Subacute combined degeneration is defined as the demyelination of the posterior and lateral columns of the cervical and thoracic region and is most commonly caused by vitamin B12 deficiency [7]. Its symptoms include deficits in vibration, proprioception, fine touch, and muscle weakness that may or may not be reversible. The neurologic findings may not often be subtle. Patients can have profound weakness and may experience sensation changes and deficits in the distal extremities. Some patients may also show signs that raise concerns for dementia, especially the elderly population. MRI is the modality of choice when evaluating a patient for subacute combined degeneration. The imaging usually reveals symmetric bilateral high signal within the dorsal columns without post-contrast enhancement. This signal has been previously described as the inverted "V" sign [8]. The signal changes typically begin in the upper thoracic region, with an ascending or descending progression. Other etiologies of these radiographic findings include other nutritional or metabolic deficiencies/toxicities such as copper-deficiency myeloneuropathy, vitamin E deficiency, and methotrexate-induced myelopathy [9-11]. Demyelinating diseases such as multiple sclerosis and transverse myelitis can present in a similar manner, but they will enhance on post-contrast MRI. Infectious causes with similar radiographic findings include human immunodeficiency virus (HIV)-associated vacuolar myelopathy, herpes virus myelitis, and neurosyphilis (tabes dorsalis) [12]. Autoimmune etiologies include sarcoidosis. Ischemia may have a similar appearance on MRI as well. Other possible diagnoses include neoplasms such as astrocytoma and ependymoma as well as hereditary syndromes such as leukoencephalopathy and Friedreich's ataxia [13]. Lactate elevation may also look similar on imaging; however, the abnormalities seen in it also involve the cerebral white matter and the brainstem [14]. Our patient had a negative HIV test and syphilis assay, as well as normal copper and vitamin E levels. There were no signs or symptoms related to other etiologies for his subacute combined degeneration.

Pernicious anemia

The most common cause of severe B12 deficiency is pernicious anemia. It is characterized by autoimmune gastritis resulting in parietal cell destruction [15]. These cells release intrinsic factor, which assists in the absorption of vitamin B12. Parietal cells also secrete hydrochloric acid, which aids in the release of B12 from ingested food. Patients with this autoimmune gastritis tend to overproduce gastrin to stimulate parietal cell acid and intrinsic factor secretion. Pernicious anemia can be associated with a chronic *Helicobacter pylori* *(H. pylori) *infection [16]. 

In this case, the patient had parietal cell and intrinsic factor antibodies and elevated gastrin levels consistent with pernicious anemia. His proton pump inhibitor was stopped to aid in B12 absorption. Pending workup for the patient included an evaluation for *H. pylori* infection as a possible etiology for his pernicious anemia. However, usually by the time of patients' presentation, the alkaline environment that is created by chronic gastritis eradicates the suitable environment for *H. pylori* bacteria [17]. Treatment for pernicious anemia includes adequate supplementation of vitamin B12 with or without iron supplementation depending on the extent of anemia present. Megaloblastic anemia, and neurologic manifestations in most cases, can be reversed with lifelong treatment.

## Conclusions

Clinicians should measure methylmalonic acid and homocysteine levels in patients if there is a suspicion for vitamin B12 deficiency despite normal B12 levels, especially if the B12 level is borderline low-normal. This deficiency can be seen in all age groups and can cause neurologic findings on exams and imaging, which can become permanent if left untreated. Once the diagnosis is confirmed, patients will often require lifelong vitamin B12 repletion. H_2_ blockers and proton pump inhibitors could worsen pernicious anemia by further reducing gastric production and intrinsic factor release and hence should be discontinued immediately.
